# No Associations Between Serum Lipid Levels or HOMA-IR and Asthma in Children and Adolescents: A NHANES Analysis

**DOI:** 10.4274/jcrpe.galenos.2019.2018.0098

**Published:** 2019-09-03

**Authors:** Min Lu, Beirong Wu, Rong Qiao, Haoxiang Gu, Ying Din, Xiaoyan Dong

**Affiliations:** 1 Shanghai Jiao Tong University, Children’s Hospital of Shanghai (Jing An Branch), Department of Pulmonary Medicine, Shanghai, China; 2 Shanghai Jiao Tong University, Children’s Hospital of Shanghai, Department of Outpatient, Shanghai, China

**Keywords:** Asthma, cholesterol, insulin resistance, lipid, lipoprotein, NHANES, obesity, wheezing

## Abstract

**Objective:**

Studies have reported inconsistent results on the associations between lipids and insulin resistance (IR) and asthma. The purpose of this study was to examine the associations between abnormal serum lipid levels and homeostatic model assessment-IR (HOMA-IR) and the presence of current asthma in children and adolescents.

**Methods:**

The United States National Health and Nutrition Examination Survey database from 1999 to 2012 was randomly searched for children (aged 3-11 years) and adolescents (aged 12-19 years) with and without asthma and with complete demographic and clinical data of interest. Logistic regression analyses were performed to examine associations between abnormal serum lipids, glucose and HOMA-IR and the current presence of asthma.

**Results:**

The data of 11,662 children (3 to 11 years of age) and 12,179 adolescents (12 to 19 years of age) were included in the analysis. The study group included 3,703 participants with asthma and 20,138 participants without asthma. The prevalence of self-reported current asthma was higher among participants aged between 3-11 years (52.9%) than among those aged between 12-19 years (50.7%). Multivariate analyses, after adjusting for sex, race, income-to-poverty ratio, low birth weight, prenatal maternal smoking, tobacco exposure, C-reactive protein level and body mass index Z-score, revealed no associations between elevated fasting plasma glucose, reduced high-density lipoprotein cholesterol, elevated low-density lipoprotein cholesterol, total cholesterol, triglycerides and HOMA-IR and the presence of current asthma in children or adolescents.

**Conclusion:**

In this cross-sectional study, no association was found between abnormal serum lipids or HOMA-IR and the presence of current asthma in children or adolescents.

**What is already known on this topic?**

Being overweight in childhood is associated with an increased risk for development of allergic disease. A link has been shown between elevated lipid levels and the development of asthma/wheezing in children and adults. Hyperinsulinemia may be associated with the development of asthma.

**What this study adds?**

Multivariate analyses found no associations between reduced high-density lipoprotein cholesterol, elevated low-density lipoprotein cholesterol, total cholesterol and triglycerides and the presence of asthma in children or adolescents. Multivariate analyses found no associations between elevated fasting plasma glucose and the presence of asthma in children or adolescents. Multivariate analyses found no associations between homeostatic model assessment-insulin resistance and the presence of asthma in children or adolescents.

## Introduction

Asthma is primarily a disease of childhood and its increasing prevalence, beginning in the 1980s, has been referred to as an asthma epidemic (1). It has been estimated that the prevalence of asthma in 1980 was 3.6%, increased to 7.5% in 1995 and further increased to 9.3% in 2010. Since 2010, the overall prevalence of childhood asthma has remained unchanged or has decreased slightly (2).

While a number of risk factors are associated with the development of childhood asthma, the condition has been linked especially to obesity and metabolic syndrome (3,4,5). Being overweight in childhood has also been associated with an increased risk of the development of allergic disease (6). In addition, increasing attention has been given to the association between hypercholesterolemia and obesity, as well as that between hypercholesterolemia and obesity with airway hyper-responsiveness, suggesting a potential role of cholesterol and lipid homeostasis in lung physiology and asthma (7,8,9,10,11,12,13,14). Rastogi et al (15) have also suggested that hyperglycemia and hyperinsulinemia may result in airway hyper-responsiveness.

Results of a number of studies have also linked elevated lipid levels with the development of asthma/wheezing in children and adults (1,2,3,4,5,16). However, the results are inconsistent with those of other studies that show no association (12,13,17), or even a negative association, between elevated lipids and asthma/wheezing (11). The reported associations between asthma and lipid levels and insulin resistance (IR) appear to be independent of body mass index (BMI) (3). As such, dyslipidemia and hyperinsulinemia, precursors to cardiovascular disease and diabetes, may also be associated with the development of asthma and confound its epidemiologic link to obesity (3,5,18). The results of most studies, however, have been limited by a cross-sectional study design and a wide range of subjects studied.

Thus, the purpose of the current study was to use a national population-based database to examine the associations between lipid levels and IR and the presence of current asthma in children and adolescents.

## Methods

### Data Source

The United States National Health and Nutrition Examination Survey (NHANES) is an ongoing cross-sectional health survey that represents the non-institutionalized population of the United States. The program uses a complex, multistage design to collect and analyze data representative of different geographic regions. The NHANES data are collected through a combination of interviews and physical examinations of participants by highly trained personnel. The survey is administered by the National Center for Health Statistics (NCHS) of the Centers for Disease Control and Prevention (CDC). Further information about the NHANES program is available at the NHANES website: https://www.cdc.gov/Nchs/Nhanes/about_nhanes.htm

Detailed information about NHANES data collection methods is available at https://wwwn.cdc.gov/Nchs/Nhanes/2003-2004/L13_C.htm and https://wwwn.cdc.gov/Nchs/Nhanes/2003-2004/L13AM_C.htm

The survey protocol and data collection methods for this present study were approved by the NHANES Institutional Review Board (IRB), and the NCHS Research Ethics Review Board (ERB) (Protocol#98-12, Protocol#2005-06, and Protocol #2011-17). All of the NHANES data were de-identified and analysis of the data by independent researchers does not require IRB approval or subject informed consent.

### Study Population

Data from seven cycles of the NHANES, conducted during the period 1999-2012 were used. The data of children (aged 3-11 years) and adolescents (aged 12-19 years) with complete demographic and laboratory data, as well as that of other variables of interest, were included in the analysis. Exclusion criteria were: 1) diagnosis of diabetes mellitus (defined as a self-report of having been told by a doctor or health professional that the subject had diabetes or sugar diabetes, or currently taking diabetic pills or insulin); 2) pregnancy; 3) being underweight, defined as a BMI <5^th^ percentile for age and sex (19,20).

### Dependent Variables (Y)

The primary outcome of the analysis was the presence of current asthma or wheezing (21). Current asthma was defined as those who reported ever being told that they had asthma and who had an asthma attack in the past year (https://wwwn.cdc.gov/Nchs/Nhanes/2007-2008/MCQ_E.htm). Wheezing was defined as wheezing or whistling in the chest in the course of the past year (https://wwwn.cdc.gov/Nchs/Nhanes/2003-2004/RDQ_C.htm).

### Independent Variables (X)

The NHANES dataset provided laboratory results of total cholesterol (TC), triglycerides (TG), high-density lipoprotein (HDL) cholesterol, low-density lipoprotein (LDL) cholesterol and fasting plasma glucose (FPG) levels. Of these, TG, LDL cholesterol and FPG were measured only in subsamples (adolescents), while TC and HDL cholesterol were measured in all participants. Homeostatic model assessment-IR (HOMA-IR) was calculated using the equation: fasting glucose (mg/dL) × fasting insulin (pmol/L) /405 /6 (22,23,24) only in subsamples. The cutoff values for abnormal lipid and FPG levels, and HOMA-IR were: ≥170 mg/dL for elevated TC; ≤45 mg/dL for low HDL cholesterol; ≥110 mg/dL for elevated LDL cholesterol; ≥75 and ≥90 mg/dL for elevated TG for participants ≤9 years old and >10 years old, respectively; ≥100 mg/dL for abnormal FPG; ≥3.0 for abnormal HOMA-IR, as reported by the expert panel of the United States National Heart, Lung, and Blood Institute (25) and used in prior investigations (15,26).

### Covariates (Potential Confounders)

Demographic data examined as potential confounders included age, sex, family income-to-poverty ratio, prenatal maternal smoking, birth weight (low birth weight or not), and C-reactive protein (CRP) level. Age- and sex-specific BMI percentiles and BMI Z-scores were determined according to the 2000 CDC growth charts using a CDC SAS program www.cdc.gov/nccdphp/dnpao/growthcharts/resources/sas.htm (6).

Tobacco exposure was defined by a “yes” response to the questions: “Have you ever tried cigarette smoking, even 1 or 2 puffs?” or “Does anyone who lives here smoke cigarettes, cigars, or pipes anywhere inside this home?”

To estimate physical activity before the year 2007, we summed the product of weekly time spent in each activity reported by the participant multiplied by the metabolic equivalent of task (MET) value for that activity yielding a MET-h index. One MET is the energy expenditure of 1 kcal/kg body weight per hour. For cycles after 2007, the physical activity questionnaire was changed. We estimated weekly MET-h for moderate and vigorous activities from questions asking participants about their participation in moderate and vigorous activities, the number of days per week engaged in these activities, and the number of minutes engaged in these activities on a typical day (27).

### Statistical Analysis

Differences in categorical variables between participants with and without asthma were determined using the Rao-Scott chi-square test and differences of continuous variables between groups were examined using the Complex Samples General Linear Model. Demographic data and baseline characteristics are expressed as mean±standard error for continuous variables, and unweighted counts (weighted %) for categorical variables. Univariate logistic regression analyses were performed to determine the association between serum lipids, glucose, HOMA-IR and current asthma. Extended-model approaches were used for covariate adjustment: Model 1 = gender, race, poverty income ratio, low birth weight (children only), prenatal maternal smoking (children only), tobacco exposure, and physical activity (adolescents only); Model 2 = Model 1 + CRP; Model 3 = Model 2 + BMI-Z-score. Participants with missing data of any covariates were not included in the regression analyses. All analyses included NHANES Medical Examination Center (MEC) sample weight or fasting subsample weight, stratum and primary sampling units per recommendations from the NCHS, to address oversampling, non-response, non-coverage and to provide nationally representative estimates. All statistical assessments were 2-sided and evaluated at the 0.05 level of significance. Statistical analyses were performed using the statistical software package SPSS complex sample module version 22.0 (IBM Corp, Armonk, NY, USA).

## Results

A total of 26,158 participants aged between 3 and 19 years were identified in the NHANES 1999-2012 cycle. Participants with diabetes (n=87), who were pregnant (n=115), or who had a BMI Z-score less than the 5th percentile (n=2,115) were excluded from the analysis, leaving 23,841 participants as the final sample.

This final eligible population included 20,138 participants without asthma and 3,703 participants with asthma, as shown in Table 1. The majority of participants were male (50.3% *vs* 55.1%, respectively), white (58.3% *vs* 58.6%, respectively), with a median income-to-poverty ratio (76.1% *vs* 75.2%, respectively), of normal birth weight (89.4% *vs* 87.3%, respectively), and with no tobacco exposure (69.7% *vs* 65.5%, respectively). The prevalence of current asthma was greater among participants aged between 3-11 years (52.9%) than among those aged between 12-19 years (50.7%). Using NHANES MEC sample weights, the analytic sample size (n=23,841) was equivalent to a population-based sample size of 65,644,773 participants (55,246,119 without asthma and 10,398,654 with asthma). Significant differences were found in sex, race, low birth weight, prenatal maternal smoking, tobacco exposure, CRP level, and BMI Z-score between groups (p<0.05).

As shown in Table 2, TC, HDL and non-HDL lipids were not associated with current asthma among children aged 3-11. In all multivariate analyses, no association was found between serum lipids and asthma after adjustment for demographic characteristics and smoking (Model 1). Addition of CRP level (Model 2), and of BMI Z-score to the analysis (Model 3) did not change the outcome (Table 3).

Univariate logistic regression showed that lower HDL [odds ratio (OR)=1.229, 95% confidence interval (CI): 1.063 to 1.421], elevated TG (OR=1.246, 95% CI: 1.013 to 1.533) and abnormal HOMA-IR (OR=1.370, 95% CI: 1.077 to 1.742) were significantly associated with higher risk of asthma in adolescents. However, after adjusting for sex, race, poverty income ratio, tobacco exposure, and physical activity, the association between asthma and HDL (OR=1.189, 95% CI: 0.992 to 1.424), TG (OR=1.161, 95% CI: 0.908 to 1.484), and HOMA-IR (OR=1.243, 95% CI: 0.950 to 1.628) became non-significant (Table 4). Again, no significant associations between serum lipid, glucose, HOMA-IR and asthma were found (Model 2 and Model 3).

## Discussion

This study was based on the NHANES database to examine the relationships between lipids and IR with the presence of current asthma in children and adolescents. Although some associations were found in univariate analysis, after controlling for confounders, multivariate analysis found no associations between lipid levels or IR and asthma in children or adolescents. Consistent with the findings of the present cross-sectional study, two case-control studies had reported no associations of asthma with lipids and IR in adults (12,13).

The potential link between obesity, diabetes and asthma has been referred to as “metabolic asthma” (3). One hypothesis is that the dysfunction of metabolic pathways that are present in obesity and diabetes exerts a direct and negative influence on the immune system, thereby affecting both adaptive and innate immunity and subsequently increasing the risk of asthma (3). Furthermore, an *in vitro* study implied that dysregulation of cholesterol transport in human airway smooth muscle cells may be important in the pathogenesis of asthma (14).

Yiallouros et al (16,18) have performed a series of studies examining the relationship between lipids and asthma in children and adolescents. In a cohort of 3,982 children from Cyprus, the authors found that low HDL cholesterol in childhood (11-12 years of age) was associated with the development of asthma in adolescence (age 15-17 years) (18). Utilizing a case-control design, these same authors found that adolescent asthma was associated with low serum HDL cholesterol levels independent of HDL levels in childhood (16). Furthermore, in a cohort of children from Cyprus, Yiallouros et al (28) found that two single nucleotide polymorphisms (SNPs) in different genetic loci were associated with both wheezing and HDL cholesterol levels, while the association between these two SNPs and asthma remains to be investigated.

Unlike most studies that did not distinguish lipid particles of different sizes, Scichilone et al (29) examined the associations between asthma and LDL subclasses in adults in a case-control study, and they found that asthma was associated with smaller LDL particles with a proinflammatory property. In addition, Barochia et al (30), in a case-control study, found that serum levels of large HDLNMR particles are positively correlated with forced expiratory volume in 1 second (FEV1) in adult patients with atopic asthma.

Two cross-sectional studies examined associations between obesity and lipids, respectively, with asthma based on the NHANES database (11,21). Visness et al (21) evaluated the association between obesity and atopic and non-atopic asthma in children and adolescents (aged 2-19) using the NHANES database (1999-2006). They found that obesity was significantly associated with current asthma among children and adolescents (OR=1.68), and that the association was stronger in non-atopic (OR=2.6) than atopic (OR=1.34) children and adolescents (21). Moreover, Fessler et al (11) examined 7005 participants ≥6 years of age who participated in the NHANES 2005 to 2006 survey, and found that serum TC and non-HDL cholesterol were negatively associated with asthma. However, the authors noted that the association was chiefly due to the strong relationship with lipid metabolism previously found in Mexican American individuals (11).

The discussion of this topic would not be complete without drawing attention to studies that *did not* find an association between lipids and asthma. Recently, Fang et al (17) compared the lipid profiles of obese asthmatic children with those of non-obese asthmatic children. The results showed that none of the asthmatic children had hypercholesterolemia and hypertriglyceridemia and that there were no differences in apo-A1 and apo-B between any of the BMI groups, nor were there differences in LDL levels (17). In a longitudinal study that followed children from birth to eight years of age, Murray et al (6) reported that although being overweight was associated with increased risk of allergic disease and wheezing, the strength of the association varied with sex, age and atopic phenotype.

### Study Limitations

There are a number of limitations to this study that potentially may have affected the results. Like most studies examining this topic, this was a cross-sectional study and therefore temporal relations and causation cannot be determined. Body composition changes during growth and hormonal expression after puberty may influence the association between adiposity and asthma, particularly among girls. The study population is restricted to a non-institutionalized population in the NHANES database, which would likely cause under-representation of severe asthma patients who were hospitalized. Data on puberty were not available in the NHANES database. Due to the lack of certain other data, we were not able to control for other potential confounding factors such as poor asthma control, lung function and diet. Inaccurate reporting or recall bias may have occurred, because NHANES surveys are based on individual or parent interviews and questionnaires. On the other hand, data from NHANES are comprehensive and nationally representative, drawing from a large and diverse sample of participants of the population of the United States. Therefore, the findings are likely to reflect the overall United States population.

## Conclusion

The results of this population-based cross-sectional study did not show an association between lipids or IR and the presence of childhood asthma. Further studies are necessary to fully understand the associations between lipids and IR and asthma.

## Figures and Tables

**Table 1:**
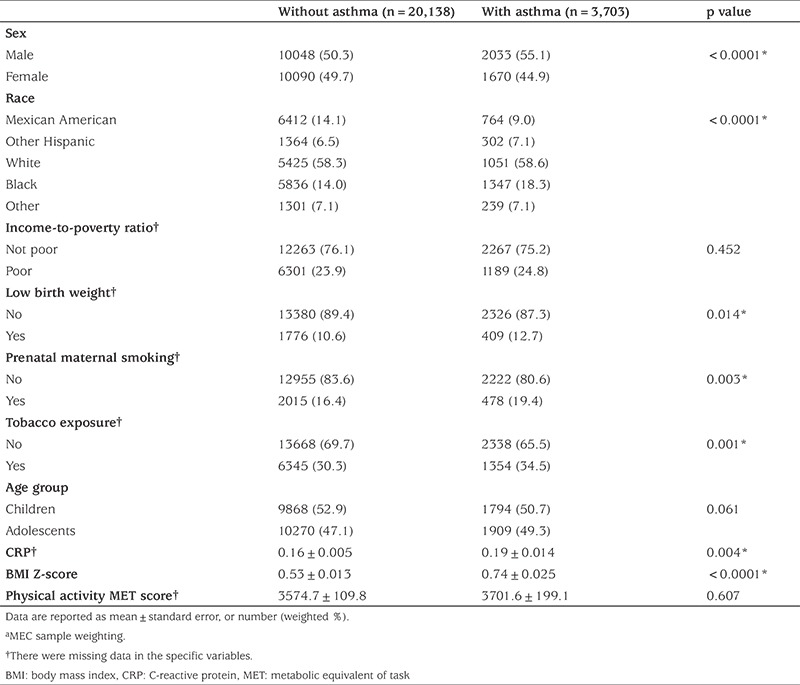
Demographic and basic characteristics of participants aged 3 to 19 years with and without asthma from NHANES 1999-2012 (unweighted n=23,841; weighted n=65,644,773^a^)

**Table 2:**
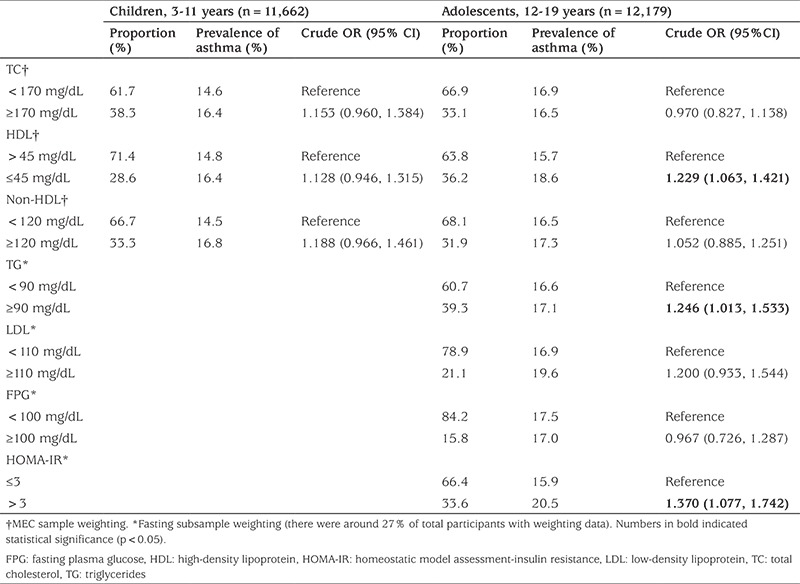
Univariate logistic regression analysis of associations between current asthma and serum lipids, glucose, and homeostatic model assessment-insulin resistance

**Table 3:**
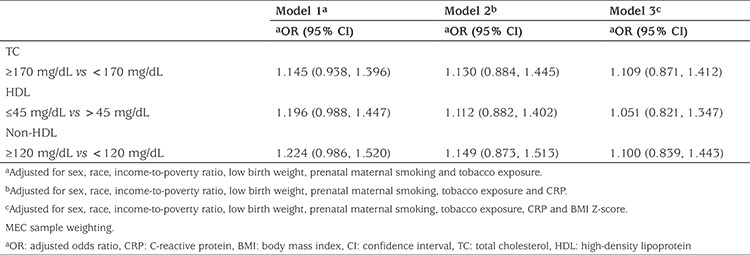
Multivariate logistic regression of the association between serum lipids and asthma in children (n=11,662)

**Table 4:**
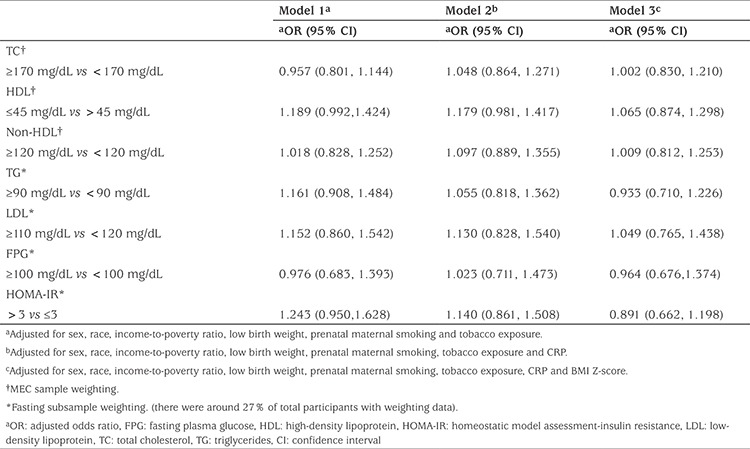
Multivariate logistic regression of the association between serum lipids, glucose and homeostatic model assessment-insulin resistance and asthma in adolescents (n=12,179)
